# Construction of a tunable promoter library to optimize gene expression in *Methylomonas* sp. DH-1, a methanotroph, and its application to cadaverine production

**DOI:** 10.1186/s13068-021-02077-8

**Published:** 2021-12-04

**Authors:** Hyang-Mi Lee, Jun Ren, Myeong-Sang Yu, Hyunjoo Kim, Woo Young Kim, Junhao Shen, Seung Min Yoo, Seong-il Eyun, Dokyun Na

**Affiliations:** 1 Department of Biomedical Engineering, Chung-Ang University, 84 Heukseok-ro Dongjak-gu, Seoul, 06974 Republic of Korea; 2 Department of Life Science, Chung-Ang University, Seoul, 06974 Republic of Korea

**Keywords:** *Methylomonas* sp. DH-1, Promoter library, Gene expression optimization, Cadaverine

## Abstract

**Background:**

As methane is 84 times more potent than carbon dioxide in exacerbating the greenhouse effect, there is an increasing interest in the utilization of methanotrophic bacteria that can convert harmful methane into various value-added compounds. A recently isolated methanotroph, *Methylomonas* sp. DH-1, is a promising biofactory platform because of its relatively fast growth. However, the lack of genetic engineering tools hampers its wide use in the bioindustry.

**Results:**

Through three different approaches, we constructed a tunable promoter library comprising 33 promoters that can be used for the metabolic engineering of *Methylomonas* sp. DH-1. The library had an expression level of 0.24–410% when compared with the strength of the *lac* promoter. For practical application of the promoter library, we fine-tuned the expressions of *cadA* and *cadB* genes, required for cadaverine synthesis and export, respectively. The strain with P_rpmB_-*cadA* and P_DnaA_-*cadB* produced the highest cadaverine titre (18.12 ± 1.06 mg/L) in *Methylomonas* sp. DH-1, which was up to 2.8-fold higher than that obtained from a non-optimized strain. In addition, cell growth and lysine (a precursor of cadaverine) production assays suggested that gene expression optimization through transcription tuning can afford a balance between the growth and precursor supply.

**Conclusions:**

The tunable promoter library provides standard and tunable components for gene expression, thereby facilitating the use of methanotrophs, specifically *Methylomonas* sp. DH-1, as a sustainable cell factory.

**Graphical Abstract:**

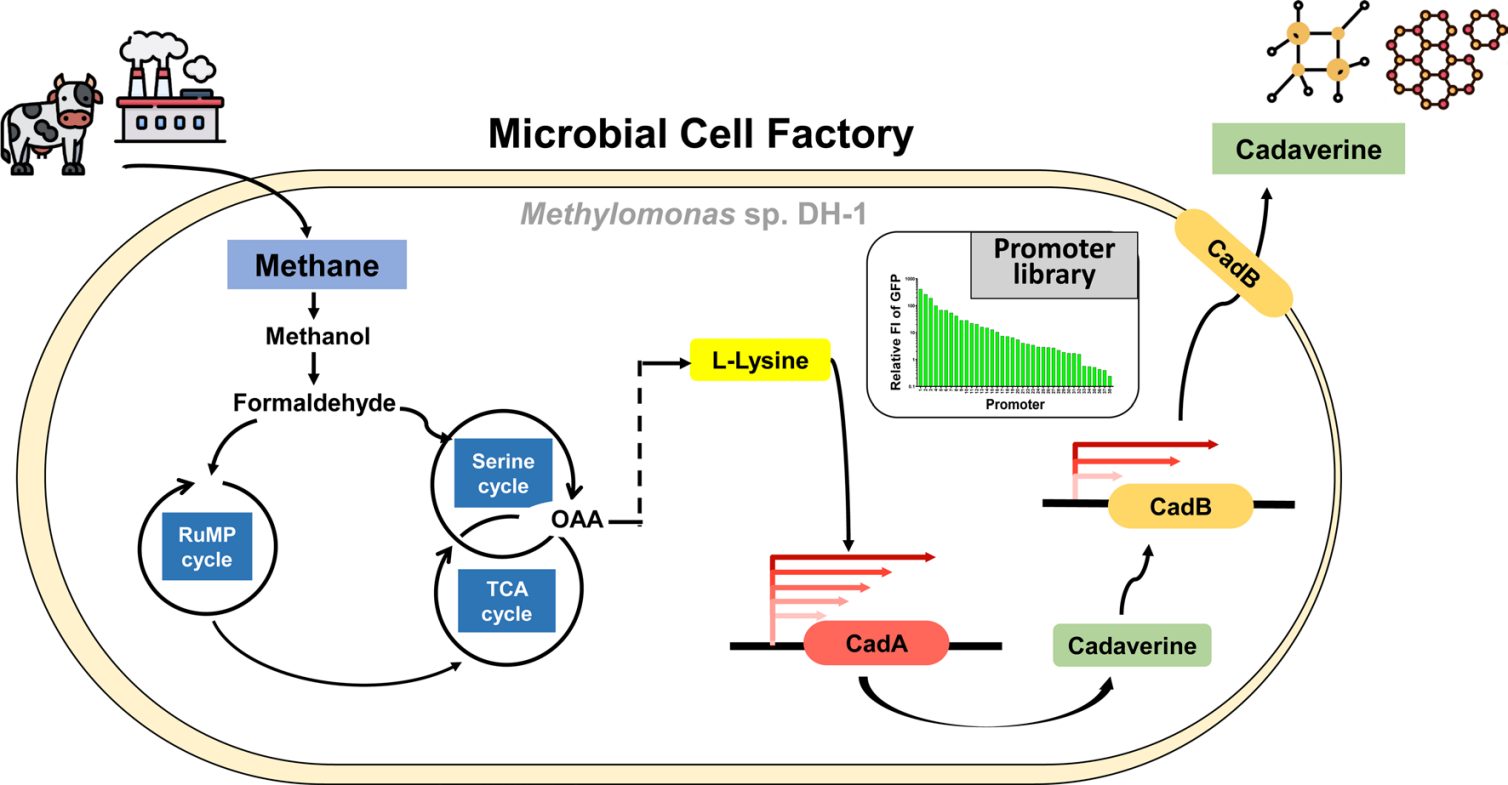

**Supplementary Information:**

The online version contains supplementary material available at 10.1186/s13068-021-02077-8.

## Background

Gene expression is a fundamental process of living organisms, specifically for processes, such as growth, metabolism, homeostasis, differentiation, and reproduction [1–3]. It has therefore been evolutionarily optimized for robust cellular functions. In cell engineering fields, such as synthetic biology and metabolic engineering, gene expression is fine-tuned for the robust operation of artificially designed genetic systems and enhanced production of a desired substance via manmade metabolic pathways [4–7].

In metabolic engineering, the overexpression of enzyme genes may fail to increase the production yield because cellular resources are excessively consumed for enzyme synthesis, resulting in decreased cell growth and thereby a decreased yield. In addition, the non-optimized expression of enzyme genes may facilitate the accumulation of toxic intermediate metabolites, which decreases the production yield. To resolve this problem, promoters with different strengths are widely employed to fine-tune the expression levels of enzyme genes [8, 9].

*Methylomonas* sp. DH-1 is a newly isolated type I methanotrophic bacterium [10]. Its genomic and transcriptomic analyses have revealed that it possesses many genes that are involved in methane metabolism and secondary metabolite biosynthetic pathways, including the tricarboxylic acid (TCA) cycle, the ribulose monophosphate (RuMP) cycle, the Embden–Meyerhof–Parnas (EMP) pathway, the pentose phosphate (PP) pathway, the Entener–Doudoroff (EDD) pathway, and the methylerythritol 4-phosphate (MEP) pathway [10, 11]. Interestingly, the *Methylomonas* sp. DH-1 genome also contains a group of genes related to conventional type II methanotrophic metabolic pathways, such as tetrahydromethanopterin and tetrahydrofolate pathways [11]. Because of its high versatility, *Methylomonas* sp. DH-1 is a promising biocatalyst for efficiently converting methane into a variety of value-added products. As a result, the bacterium is used as a cell factory for producing various chemicals, such as methanol (1.34 g/L) [12], 2-propanol (0.424 g/L) [13], succinate (0.195 g/L) [14], d-lactate (1.19 g/L) [15], and acetone (16.62 mM) [16].

Despite the versatility of *Methylomonas* sp. DH-1, however, the lack of well-established genetic engineering tools hampers its wide use as a cell factory platform. In an effort to facilitate genetic engineering of *Methylomonas* sp. DH-1, several synthetic tools for genetic manipulation have been developed over the past few years [17–19]; however, there is an increased demand to develop a gene expression toolkit for the optimized metabolic engineering of *Methylomonas* sp. DH-1. A physicochemical transformation method using various chemicals (such as RbCl, LiAc, CsCl, and MgCl_2_) and nanoparticles (such as sepiolite, gold(III) chloride, and chitosan) has been developed to increase the efficiency of transformation [17]. The restriction–modification system of *Methylomonas* sp. DH-1 has also been recently identified. The identified cytosine methyltransferase has been used for DNA methylation to protect DNA sequences, which increases the transformation efficiency [18]. More recently, a protein delivery system using cell-penetrating peptides was developed for manipulating the genome because no artificial episomal plasmids for the bacterium have yet been developed [19]. An application using a delivery system was used to excise an antibiotics resistance gene from the genome truncated by loxP sites. The delivery system directly transported Cre recombinase into the cytoplasm and successfully excised the target gene from the genome. Homologous recombination for gene disruption or integration and adaptive laboratory evolution for tolerance increase have also been performed as part of the metabolic engineering of *Methylomonas* sp. DH-1 [14, 15].

In this study, we construct a library of promoters that work in *Methylomonas* sp. DH-1 and can be used as components to fine-tune its gene expression. To demonstrate the applicability of the promoter library, we fine-tuned the expressions of two genes required for the biosynthesis and export of cadaverine, *cadA* and *cadB*. Cadaverine is a bionylon monomer used for polyamide production. The developed tunable promoter library is a powerful tool for efficient expression optimization and may help advance the development of a sustainable chemical production platform via C1 assimilation.

## Results and discussion

### Construction of the promoter library for *Methylomonas* sp. DH-1

**Fig. 1:**
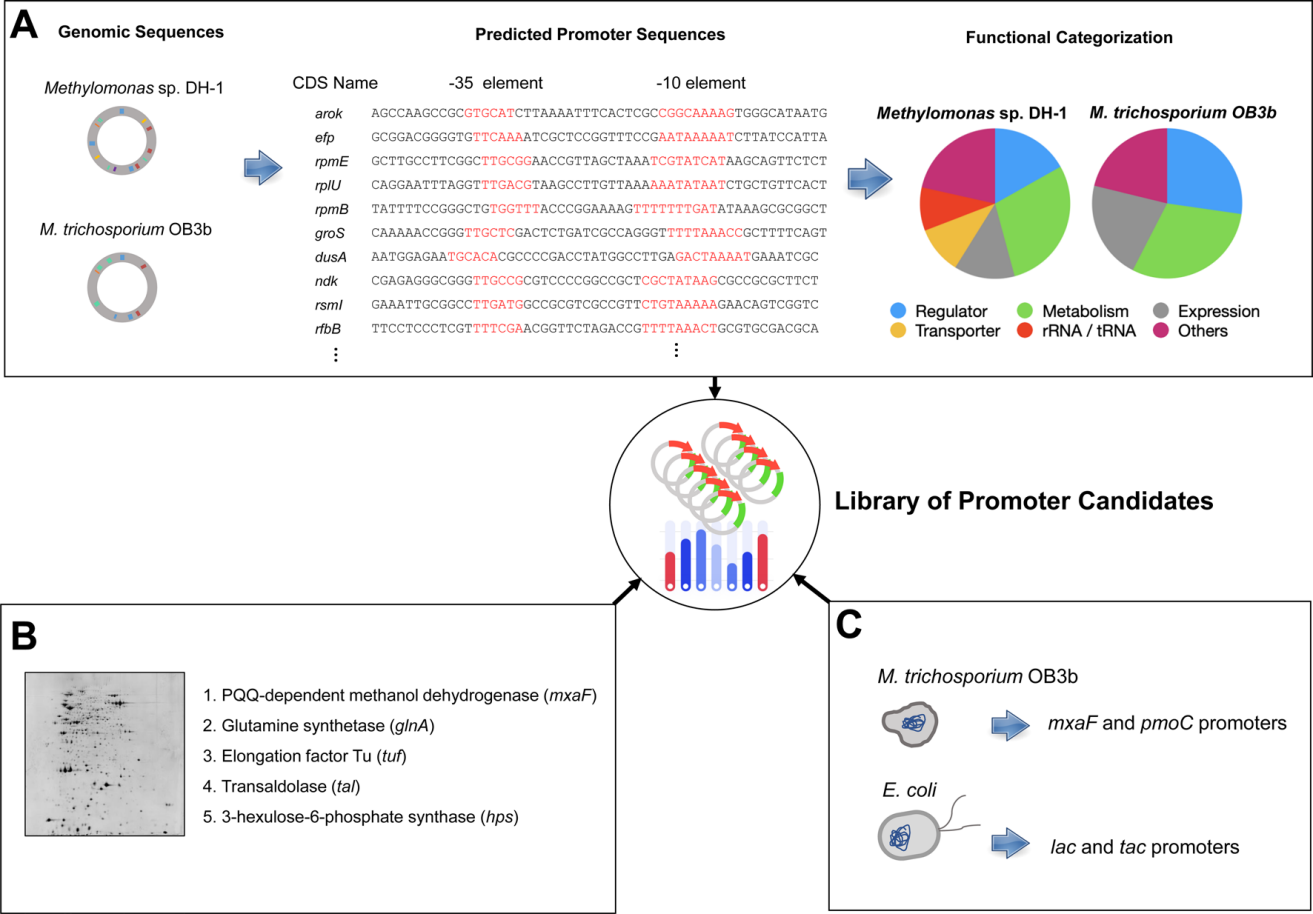
Construction of a library of promoter candidates in *Methylomonas* sp. DH-1. **A** In silico approach to predict promoters from the genomic sequences of *Methylomonas* sp. DH-1 and *M. trichosporium* OB3b and the functional classification of the coding sequences under the control of the predicted promoters. **B** 2D-PAGE approach to identify strong native promoters that allow for the overexpression of *Methylomonas* sp. DH-1. **C** Addition of four well-known promoters of *M. trichosporium* OB3b and *Escherichia coli* to the library

Among the various parameters that determine the expression level of a gene, transcription is the first step and the main target for gene regulation. As a result, promoters have been widely examined and used to control gene expression [20–22]. To identify potential promoters that can be used for gene expression optimization, we first utilized computational models to predict promoter sequence regions from the genomic sequence of *Methylomonas* sp. DH-1 [23, 24]. The computational prediction could not identify all the promoters of *Methylomonas* sp. DH-1 because the tools used have not been developed for *Methylomonas* sp. DH-1. To complement that, we also evaluated the promoters of *M. trichosporium* OB3b, a model organism of type II methanotroph [25], because *Methylomonas* sp. DH-1 also contains genes related to type II methanotroph [11] and they may share similar consensus promoter sequences. Therefore, the prediction was also performed on the genomic sequence of *M. trichosporium* OB3b to find more promoter candidates that could be applied to *Methylomonas* sp. DH-1. A total of 110 promoter candidates were predicted: 93 sequences from *Methylomonas* sp. DH-1 and 17 from *M. trichosporium* OB3b. When the predicted promoters were functionally categorized based on their downstream coding sequences, most of them can be grouped into genetic regulators, metabolism, and gene expression (Fig. 1A). Detailed information on the promoters is available in Additional files 2 and 3: Tables S1 and S2. In these tables, a promoter sequence has been defined as an upstream 100-bp sequence from the transcription start site, including -35 and -10 elements.

To find promoters with enough strength for efficient overexpression, the total cellular proteome of *Methylomonas* sp. DH-1 was quantitatively analysed by 2D gel electrophoresis (2D-PAGE) [26] (Fig. 1B; Additional file 1: Fig. S1). We chose five highly dense protein spots (indicated by arrows in Additional file 1: Fig. S1), which were then excised and digested with trypsin. The resulting peptides were analysed by matrix-assisted laser desorption/ionization–time of flight (MALDI-TOF) mass spectrometry, and the corresponding proteins were identified by peptide mass fingerprinting. The five highly expressed proteins were pyrroloquinoline quinone (PQQ)-dependent methanol dehydrogenase (encoded by the *mxaF* gene), glutamine synthetase (encoded by the *glnA* gene), elongation factor Tu (encoded by the *tuf* gene), transaldolase (encoded by the *tal* gene), and 3-hexulose-6-phosphate synthase (encoded by the *hps* gene). We extracted the 5′ upstream region of the protein-coding genes to obtain their promoter sequences, including − 35 and − 10 elements. The identified proteins and their promoter sequences are listed in Additional file 4: Table S3. Interestingly, none of the promoters identified by 2D-PAGE analysis overlapped with the promoters predicted by the computational tool.

In addition to the predicted and identified promoters, we included known promoters in our library: two *M. trichosporium* OB3b promoters (methanol dehydrogenase (*mxaF*) and methane monooxygenase (*pmoC*)) and two *E. coli* promoters (*lac* and *tac*) (Fig. 1C). Finally, we compiled a library of 119 promoter candidates to construct a tunable promoter library (Fig. 1)*.*

### Evaluation of promoter candidates in *Methylomonas* sp. DH-1

To evaluate the promoter candidates, in addition to the promoters identified from 2D-PAGE analysis and the known promoters, we randomly selected several promoters from each category. The transcriptional activities of 38 out of 119 candidate promoters in *Methylomonas* sp. DH-1 were quantitatively measured. For the strength measurement, a plasmid harbouring the green fluorescent protein (*gfp*) gene under the control of a promoter candidate was constructed.

**Fig. 2:**
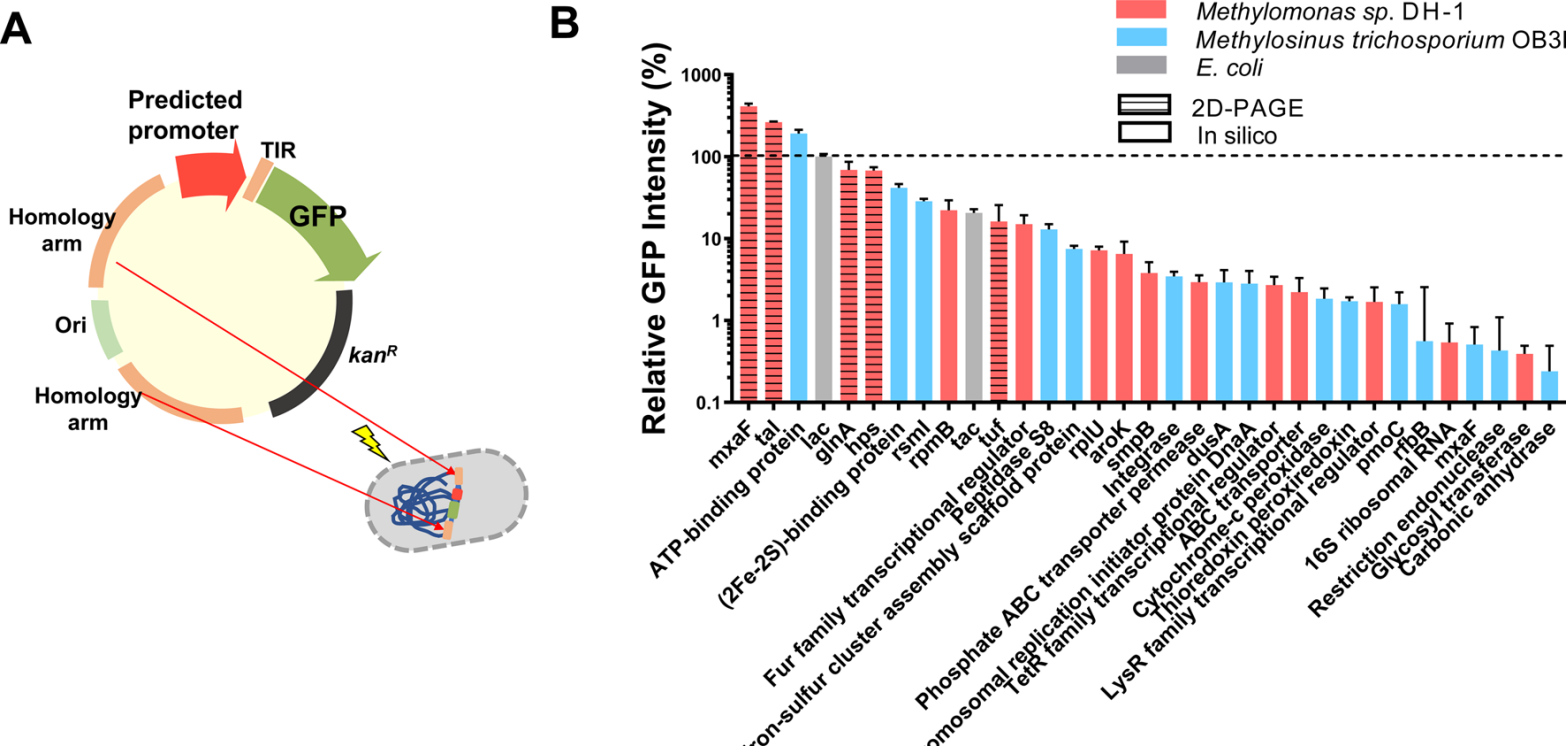
Strength of promoter candidates measured by a GFP reporter. **A** Construction of a GFP expression plasmid under the control of a predicted promoter for integration into a *Methylomonas* sp. DH-1 chromosome. **B** Promoter strength measurement in *Methylomonas* sp. DH-1 by flow cytometry. Promoters showed up to 410-fold differences in GFP expression. The strength was normalized by that of the *lac* promoter (100%). As the y-axis is drawn at log scale, five promoter candidates that showed no detectable fluorescence were excluded from the graph. Data indicate mean ± SEM (*n* = 3)

Protein production level is determined not only by promoter strength but also by translational efficiency. To exclude the effect of translation on protein production, we used the same 50-nt long UTR, SD, and GFP coding sequences, because translation initiation region (around 30-nt before and after the start codon) is a determinant of translational efficiency [27, 28]. The promoter-*gfp* gene was integrated into the non-coding region of the genome of *Methylomonas* sp. DH-1 for fluorescence measurement (Fig. 2A).

The strength of the promoters varied from 0.24% to 410% in *Methylomonas* sp. DH-1 compared with that of the *lac* promoter, which shows that the library can cover approximately 1708-fold range of expression levels in *Methylomonas* sp. DH-1 and can be used to fine-tune the gene expression. The transcriptional activities of the promoters are shown in Fig. 2B and Table 1. Five of the 38 evaluated promoters displayed no detectable fluorescence at all. We also found that the five promoters identified from 2D-PAGE analysis exhibited high transcriptional activity. In particular, the promoter of the *mxaF* gene from *Methylomonas* sp. DH-1 showed the highest expression level, indicating that this promoter can be used for the overexpression of a target gene. Because the *mxaF* gene encodes the α-subunit of methanol dehydrogenase, a key enzyme in the methanol-to-formaldehyde conversion for methane utilization, the promoter could be highly active. In contrast, the promoter strength of the *mxaF* gene from *M. trichosporium* OB3b—a strong promoter [29]—was < 1% in *Methylomonas* sp. DH-1. The GFP intensity under the control of P_tal_ showed the second-highest intensity. Typically, transaldolases function in the non-oxidative phase of the PP pathway in carbohydrate metabolism to generate nicotinamide adenine dinucleotide phosphate (NADPH) and ribose, which are essential for the biosynthesis of secondary metabolites and amino acids.

**Table 1:** Tunable promoter library for *Methylomonas* sp. DH-1

Promoter identification method	Gene name	Promoter strength (%)
In silico prediction (*Methylomonas* sp*.* DH-1)	50S ribosomal protein L28 (*rpmB*)	22.09
	Fur family transcriptional regulator	14.99
	50S ribosomal protein L21 (*rplU*)	7.16
	Shikimate kinase (*aroK*)	6.49
	SsrA-binding protein (*smpB*)	3.79
	Phosphate ABC transporter permease	2.94
	TetR family transcriptional regulator	2.7
	ABC transporter	2.21
	LysR family transcriptional regulator	1.68
	16S ribosomal RNA	0.54
	Glycosyl transferase	0.39
	Cytochrome c oxidase subunit 2	0.0
	Elongation factor P (*efp*)	0.0
	50S ribosomal protein L31 (*rpmE*)	0.0
	10 kDa chaperonin (*groS*)	0.0
In silico prediction (*M. trichosporium* OB3b)	ATP-binding protein	191.08
	(2Fe–2S)-binding protein	41.54
	Ribosomal RNA small methyltransferase I (*rsmI*)	28.42
	Peptidase S8	12.92
	Iron–sulphur cluster assembly scaffold protein	7.45
	Integrase	3.45
	tRNA-dihydrouridine(20/20a) synthase (*dusA*)	2.91
	Chromosomal replication initiator protein DnaA	2.81
	Cytochrome *c* peroxidase	1.84
	Thioredoxin peroxiredoxin	1.71
	dTDP-glucose 4,6-dehydratase (*rfbB*)	0.56
	Restriction endonuclease	0.43
	Carbonic anhydrase	0.24
	GGDEF domain-containing protein	0.0
2D-PAGE (*Methylomonas* sp. DH-1)	Methanol dehydrogenase (*mxaF*)	411.18
	Transaldolase (*tal*)	262.9
	Glutamine synthetase (*glnA*)	68.74
	3-hexulose-6-phosphate synthase (*hps*)	67.37
	Elongation factor Tu (*tuf*)	16.12
*M. trichosporium* OB3b promoters	Methane monooxygenase (*pmoC*) promoter C	1.58
	Methanol dehydrogenase (mxaF) promoter	0.51
*E. coli* promoters	*lac* promoter (without operator)	100
	*tac* promoter (without operator)	20.54

In general, there is an overall correlation between transcript level and protein level, but there are also many reports on the inconsistency of the correlation [30]. For example, highly transcribed mRNAs often failed to be highly translated [31]. To investigate if the high expression of the proteins was due to the high strength of their promoters, not high efficiency in translation, we confirmed their transcript levels by using the previous transcript data of *Methylomonas* sp. DH-1 [11]. According to the previous gene expression profile, of the five highly expressed proteins identified by the 2D-PAGE, only the transcription levels of three proteins were available (methanol dehydrogenase, transaldolase, and 3-hexulose-6-phosphate synthase). The three proteins were involved in the methanol oxidation, the PP pathway, and the RuMP cycle, respectively, and were grouped into a high transcription category (Fig. 2B). Interestingly, the GFP intensities expressed from the P_ATP-binding protein_ of *M. trichosporium* OB3b and the *lac* promoter of *E. coli* took third and fourth places, respectively. Because of the wide range of expression levels, the developed library of promoters can be utilized for fine-tuning gene expression in *Methylomonas* sp. DH-1. It should be noted that promoters may display different activity under different conditions due to regulatory effects. Thus, for the consistent measurement of promoter activity, we chose a defined expression context, such as exponential growth phase and methane as a carbon source [32], and the developed library was ensured to operate under the defined context.

### Influence of the expression level of *cadA* on cell fitness and cadaverine production

Because *Methylomonas* sp. DH-1 has various metabolic pathways, such as the RuMP pathway, the PP pathway, the EMP pathway, the TCA cycle, and the serine cycle, it has been engineered as a promising host platform for C1 microbial cell factories [12–16]. As a practical application of our tunable promoter library, we diversified the expression level of the *cadA* gene to maximize the cadaverine titre from *Methylomonas* sp. DH-1.

**Fig. 3:**
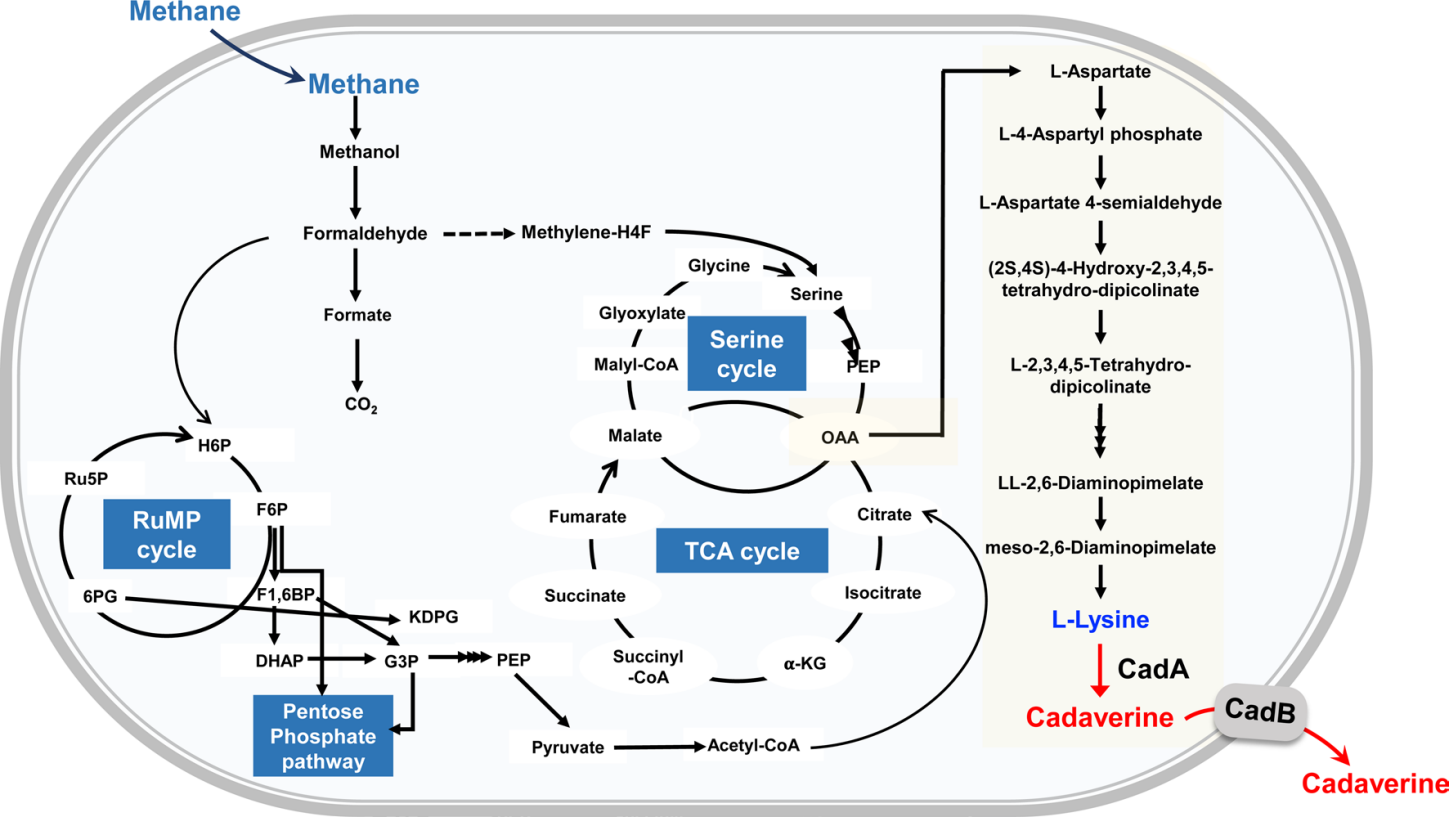
Metabolic pathways of *Methylomonas* sp. DH-1 for the production of cadaverine from methane. Reactions mediated by heterologous enzymes are shown in red. CadA, l-lysine decarboxylase; CadB, lysine/cadaverine antiporter protein

Generally, the microbial production of cadaverine, a natural nylon monomer used for polyamide production, is achieved through the overexpression of the *E. coli* lysine decarboxylase (CadA), which bioconverts lysine to cadaverine. Hence, it can be used in the chemical industry [33, 34]. The biosynthetic pathway towards lysine/cadaverine synthesis in *Methylomonas* sp. DH-1 is illustrated in Fig. 3. In native *Methylomonas* sp. DH-1, lysine synthesis involves oxaloacetate (OAA) derived from the serine and TCA cycles. However, because of the lack of the *cadA* gene, the bacterium cannot synthesize cadaverine. Thus, we introduced the *cadA* gene from *E. coli* into the genome of *Methylomonas* sp. DH-1 for cadaverine production.

Fine-tuning the CadA expression is important for efficient cadaverine production and cell fitness, because overexpressed CadA might deplete lysine, which is also used as a precursor for cell wall synthesis [35]. In addition, the highly accumulated cadaverine could inhibit the activity of CadA [36] or induce cytotoxicity. Thus, the level of CadA expression should be balanced for optimum cell growth/toxicity and cadaverine production.

**Fig. 4:**
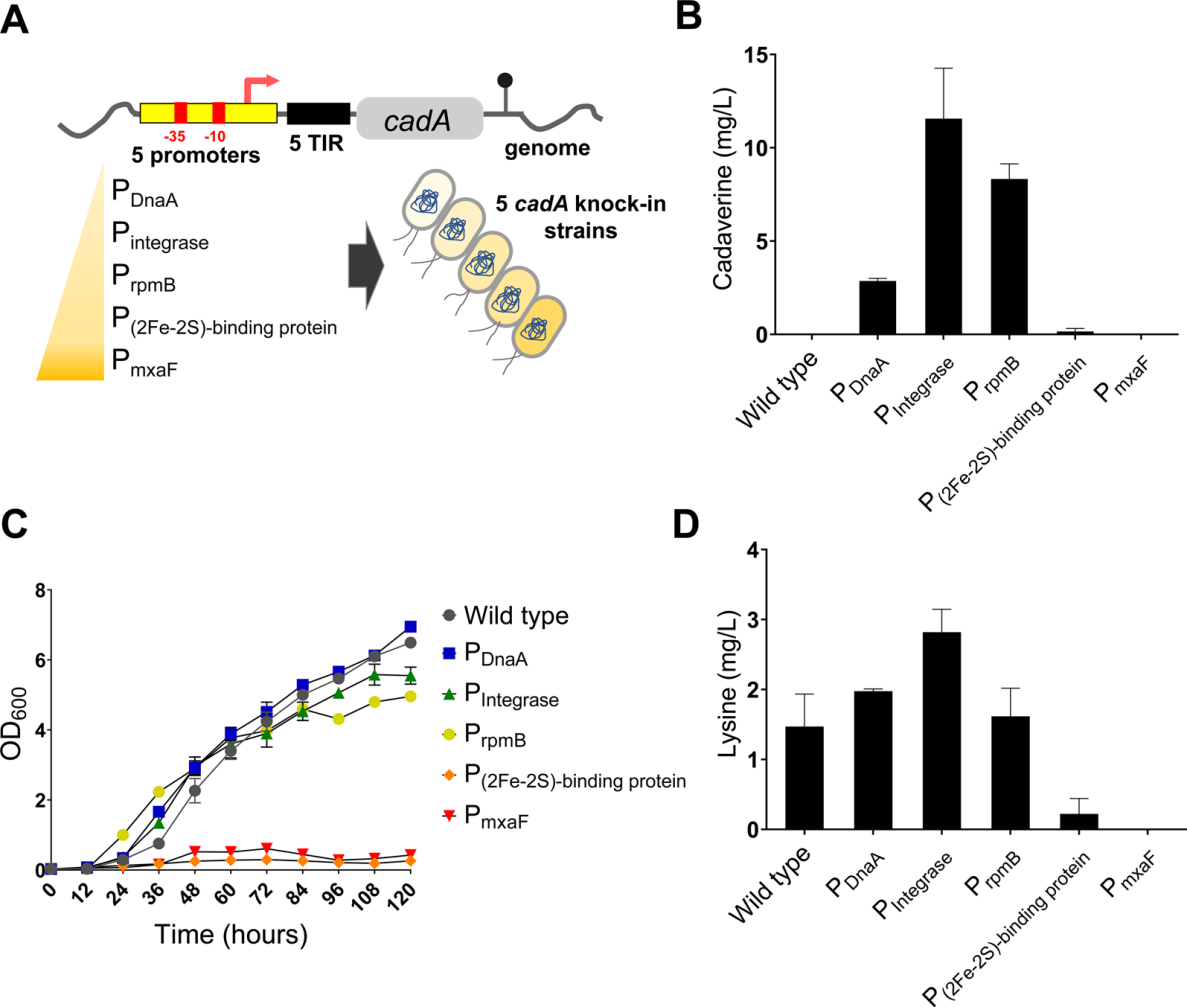
Effects of the expression level of the *cadA* gene on cell growth and lysine/cadaverine biosynthesis in *Methylomonas* sp. DH-1. **A** Genetic structure of *cadA* under the control of five promoters. Cadaverine production (**B**), growth (**C**), and lysine synthesis (**D**) of wild-type and five engineered *Methylomonas* sp. DH-1 strains in an NMS medium with a supplementation of 30% methane (v/v) as the sole carbon source. Data indicate mean ± SEM (*n* = 3)

We employed five promoters of different strengths from our library to diversify the expression level of CadA (Fig. 4A). The promoters and their relative strengths were P_DnaA_ (2.81%), P_Integrase_ (3.45%), P_rpmB_ (22.09%), P_(2Fe–2S)-binding protein_ (41.54%), and P_mxaF_ (411%). We then introduced the five constructed genes into the genome of *Methylomonas* sp. DH-1 and tested the resulting strains for the desired bioconversion in shake-flask cultures with a supplementation of 30% methane (v/v) as the sole carbon source. Therefore, we measured cadaverine and its precursor lysine titres along with cell growth. As shown in Fig. 4B, C, the CadA overexpression by two strong promoters (P_mxaF_ and P_(2Fe–2S)-binding protein_) failed to produce cadaverine and greatly retarded the cell growth (Fig. 4B, C). On the contrary, the strains expressing CadA under the control of three weeks or moderate promoters (P_DnaA_, P_Integrase_, and P_rpmB_) displayed normal growth and higher cadaverine titres of 2.85 ± 0.13, 11.55 ± 2.70, and 8.32 ± 0.81 mg/L, respectively, after 72 h of cultivation. In a previous study, the same tendency of cadaverine titre change with respect to the level of CadA had been observed in *E. coli* [37]: when CadA was highly expressed, the cadaverine titre was decreased. One plausible hypothesis for the decreased cadaverine titre is lysine depletion. As shown in Fig. 4D, when the two strong promoters were employed, the lysine titre inside *Methylomonas* sp. DH-1 was remarkably decreased. Since lysine is an important amino acid for growth, the depletion also induced cell growth defect [38], which in turn resulted in a decrease in overall cadaverine titre (Fig. 4B). Interestingly, the lysine concentrations in the strains of the three weak or moderate promoters were not statistically different from that of wild type when tested by t-test (*p*-value > 0.1), even though the lysine-consuming *cadA* gene was introduced. It is thus likely that CadA induced a metabolic flux in the lysine biosynthetic pathway by consuming lysine at an appropriate rate. Collectively, these results suggest that fine-tuning the expression of a target gene, rather than its overexpression, is a key to engineer metabolic pathways and that our tunable library can provide a genetic tool for optimization.

### Improved cadaverine production by optimizing *cadB* expression

**Fig. 5:**
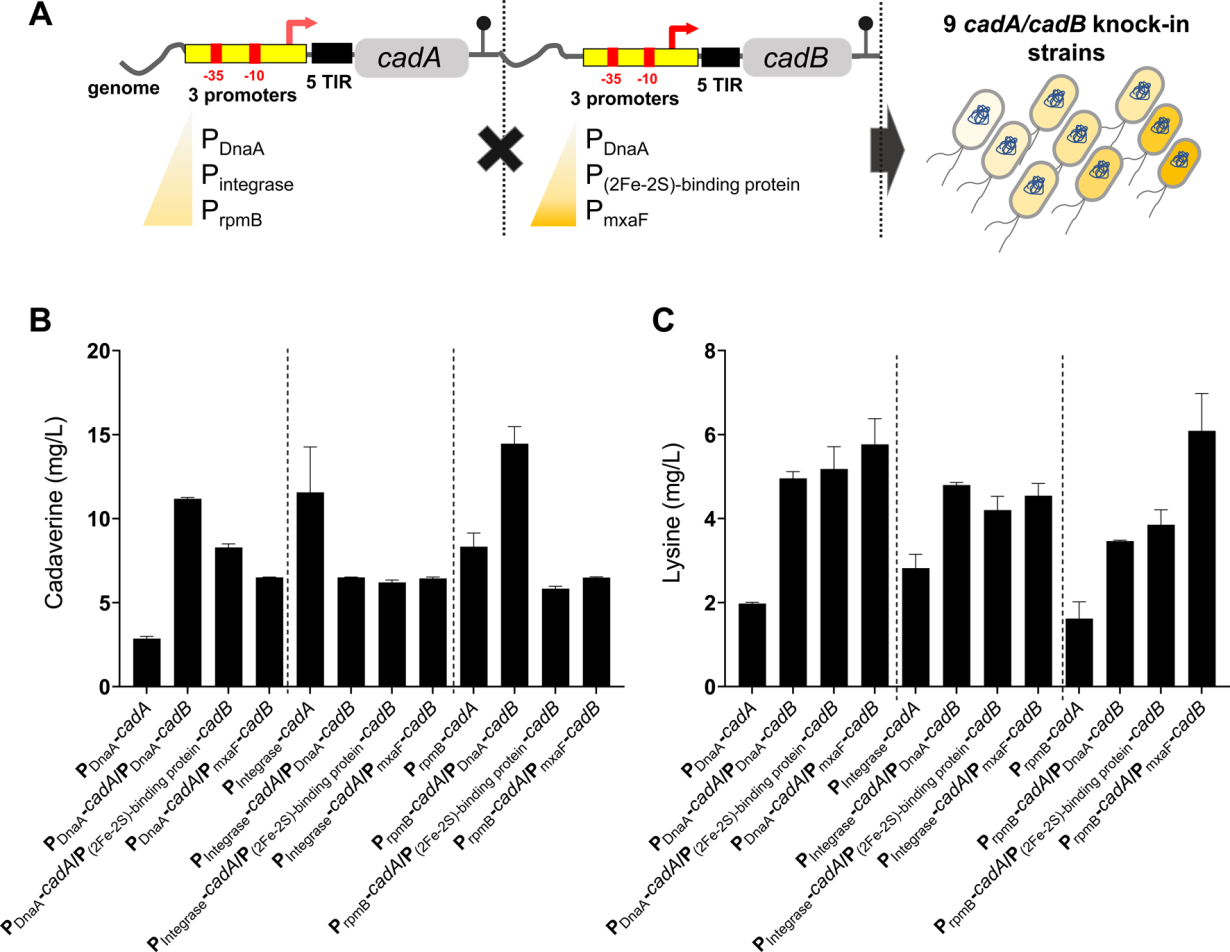
Effect of the expression level of the *cadB* gene on lysine/cadaverine biosynthesis in three selected *cadA* knock-in strains. **A** Genetic structure for *cadA* and *cadB* expression. Three different promoters (P_DnaA_, P_(2Fe–2S)-binding protein_, and P_mxaF_) were employed to diversify the expression level of *cadB*. Cadaverine (**B**) and lysine titres (**C**) in the engineered *Methylomonas* sp. DH-1 strains. Data indicate mean ± SEM (*n* = 3)

The optimization of *cadA* gene expression in *Methylomonas* sp. DH-1 using a promoter library helped enhance cadaverine production without redesigning the lysine synthetic pathway. It has been reported that the co-expression of CadA and CadB (a lysine/cadaverine antiporter) could simultaneously enhance lysine and cadaverine production because CadB exports intracellular cadaverine, a feedback inhibitor of CadA [33, 35, 36]. To further enhance cadaverine production, we introduced the *cadB* gene into three cadaverine-producing strains, P_DnaA_-*cadA*, P_Integrase_-*cadA*, and P_rpmB_-*cadA*. To investigate whether the fine-tuning of CadB gene expression could also affect cell growth and lysine/cadaverine production, we co-expressed the *cadB* gene under three different promoters (P_DnaA_, P_(2Fe–2S)-binding protein_, and P_mxaF_) and constructed nine strains in total (Fig. 5A).

**Fig. 6:**
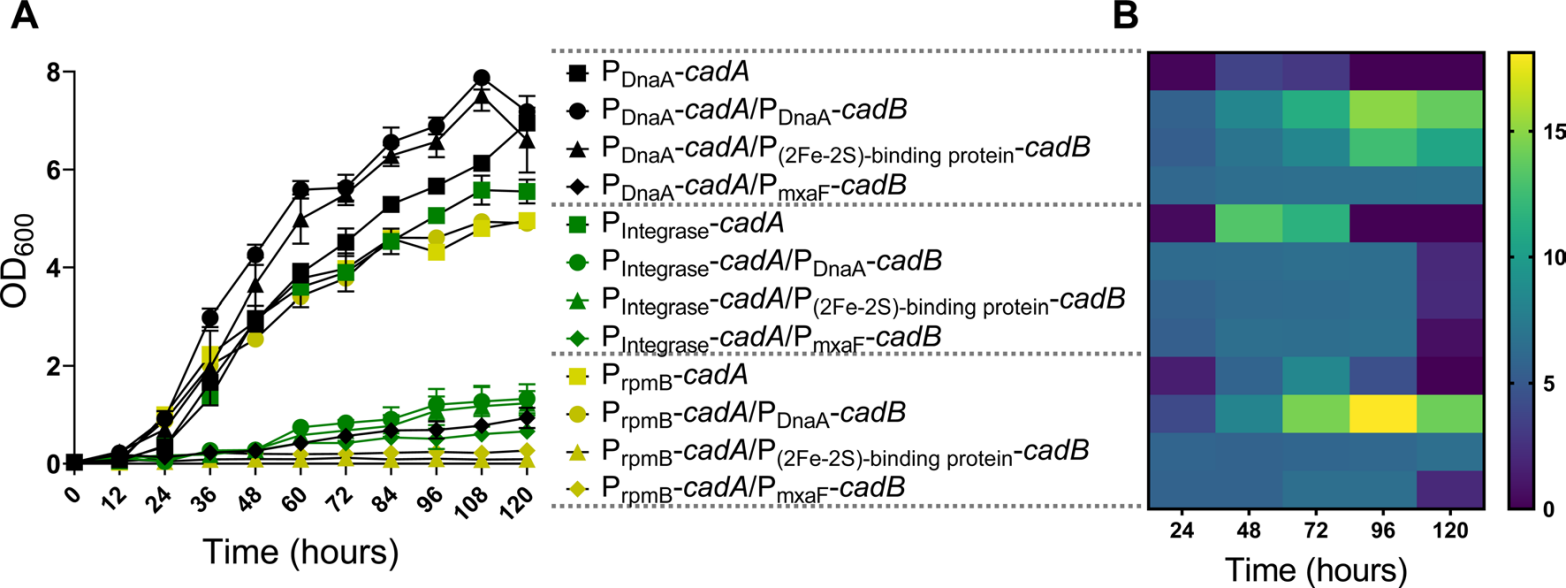
Cell growth (**A**) and cadaverine titre (**B**) over time with respect to the employed promoter for the *cadB* gene. Data indicate mean ± SEM (*n* = 3)

As shown in Fig. 5B, the highest cadaverine titres, 11.18 ± 0.07 and 14.46 ± 1.01 mg/L, were achieved from the P_DnaA_-*cadA*/P_DnaA_-*cadB* and P_rpmB_-*cadA*/P_DnaA_-*cadB* strains, respectively, after 72 h of cultivation. The cadaverine titres were improved by 3.92- and 1.73-fold compared with those of their parental strains, P_DnaA_-*cadA* and P_rpmB_-*cadA*, respectively. Interestingly, although the introduction of *cadB* increased the lysine titre in all *cadA*/*cadB* knock-in strains, its introduction into the P_Integrase_-*cadA* strain reduced cadaverine production in all promoters. To investigate the reason for the inefficient production of cadaverine in these strains, the cell growth and cadaverine production were monitored for 120 h (Fig. 6A, B). Interestingly, when *cadB* was overexpressed, the cells showed a major growth defect and decreased the cadaverine titre. Unlike the *cadA* gene, the overexpression of the *cadB* gene did not deplete the intracellular lysine concentration (Fig. 5C). One plausible explanation is that the additional overproduction of CadB consumed intracellular resources and posed a burden to the cell, thereby retarding the cell growth; however, this should be elucidated further. After 96 h of cultivation, the engineered P_rpmB_ -*cadA*/P_DnaA_-*cadB* strain afforded the maximum cadaverine titre (18.12 ± 1.06 mg/L), which was 2.18-fold higher than that (8.32 ± 0.81 mg/L) of its parental strain (P_rpmB_-*cadA* strain) and 2.78-fold higher than that (6.50 ± 0.02 mg/L) of the non-optimized *cadA/cadB* strain (P_rpmB_ -*cadA*/P_(2Fe–2S)-binding protein_-*cadB*).

## Conclusions

For the first time, a tunable library consisting of 33 promoters of different strengths was constructed for the gene expression optimization of *Methylomonas* sp. DH-1. The results showed that the fine-tuning of transcription rather than overexpression allows efficient production of recombinant proteins and regulation of metabolic pathways in cells. In future studies, an inducible system based on the tunable promoter library should be designed for more dynamic control of transcription. This system will help make methanotrophs a major sustainable platform for producing value-added products via C1 assimilation. Overall, the promoter library discussed here will facilitate the genetic manipulation of *Methylomonas* sp. DH-1 for successful implementation of methanotroph biotechnology.

## Methods

### Growth conditions

The *E. coli* DH5α strain was utilized for DNA cloning and plasmid preparation. A lysogeny broth (LB) medium, containing 10 g of tryptone, 5 g of yeast extract, and 10 g of sodium chloride per litre, was used for the cultivation of the *E. coli* strain, with appropriate antibiotics (100 μg/mL of ampicillin or 50 μg/mL of kanamycin).

*Methylomonas* sp. DH-1 (KCTC13004BP) was used as a parental strain of the engineered strains. It was cultured in a nitrate mineral salt (NMS) medium [14] supplemented with 30% (v/v) methane at 30 °C with shaking at 250 rpm. For genetic integration, the cells were transformed with plasmid-containing homology arms by electroporation [19] and grown in NMS plates with appropriate antibiotics (100 μg/mL of ampicillin and/or 10 μg/mL of kanamycin). To measure cell growth, *Methylomonas* sp. DH-1 cells were grown until the stationary phase was reached. They were then diluted to OD_600_ = 0.03 with a fresh NMS medium and 30% (v/v) methane. For repeat methane feeding, gas substitution was performed using a gas-tight syringe. The headspace was refreshed daily. Cell growth (OD_600_) was monitored for 5 days using a Hitachi U5100 UV–Vis spectrophotometer (Tokyo, Japan).

### Computational prediction of methanotroph promoters

We obtained whole genome sequences and gene annotation data of two bacteria, *Methylomonas* sp. DH-1 (CP014360.1) and *M. trichosporium* Ob3b (CP023737.1), from NCBI GenBank. From the chromosome data, we collected 100-bp upstream region from the transcription start site (TSS) of all genes as potential promoter sequences, except for the genes that produce a hypothetical protein.

To identify the methanotroph promoter, we employed two promoter prediction models: Softberry BPROM [24] and the BDGP neural network promoter prediction tool [23]. To increase the prediction accuracy, we selected the promoter regions recognized by both tools as candidates. As a result, 93 sequences from *Methylomonas* sp. DH-1 and 17 from *M. trichosporium* Ob3b were predicted as methanotroph promoter candidates.

### 2D-PAGE experiments

2D-PAGE experiments were performed as described previously [39]. The *Methylomonas* sp. DH-1 cells were suspended and mixed with a lysis buffer (8 M urea, 2 M thiourea, 40 mM Tris, 65 mM DTT, and 4% (w∕v) CHAPS). Proteins in the supernatant (200 μg) were diluted into 340 μL of a rehydration buffer (8 M urea, 2 M thiourea, 20 mM DTT, 2% (w∕v) CHAPS, 0.8% (w∕v) immobilized pH gradient (IPG) buffer, and 1% (v∕v) cocktail protease inhibitor) and then loaded onto Immobiline DryStrip gels (18 cm, pH 3–10 NL; GE Healthcare Bio-Sciences, Uppasala, Sweden). The loaded IPG strips were rehydrated, focused, and equilibrated and then transferred to 12% (w∕v) SDS–polyacrylamide gels. The 2D image was analysed using PDQuest 2D Analysis Software (BioRad). The protein spots in the 2D-gel were identified by comparing them with those of a previous report and using the *E. coli* 2D database (http://world-2dpage.expasy.org/swiss-2dpage/viewer) as a reference [26].

### Construction of plasmids for genomic integration into *Methylomonas* sp. DH-1

The pIns plasmid [19], which carries two 1-kb-long homology arms for genomic integration into the genome of *Methylomonas* sp. DH-1 and the resistance marker gene *Kan*^R^, was used to develop further plasmid versions. To measure the strength of the predicted promoters, we additionally inserted the predicted promoter sequence, a modified TIR sequence, and a *gfp* gene between the two homology arms through restriction enzyme digestion and ligation. The promoter sequences were amplified from the genomic DNA of *Methylomonas* sp. DH-1. The correct insertion was verified by DNA sequencing. The plasmid construction is shown in Fig. 2A.

To integrate lysine decarboxylase gene *cadA* for cadaverine production, the coding sequence was amplified from *E. coli* W3110 genomic DNA and cloned into the plns plasmid instead of the *gfp* gene under the control of five selected promoters. In addition, for double integration of cadaverine transport protein gene *cadB* in the background of the *cadA* knock-in cells, the homologous regions of the plns plasmid were modified to insert *cadB* at a different genomic location and the resistance gene was changed to *Amp*^R^. Then, the coding sequence of *cadB* was amplified from *E. coli* W3110 genomic DNA and cloned into the *cadA* locus under the control of three selected promoters. Each generated plasmid was integrated into a non-coding region of the *Methylomonas* sp. DH-1 chromosome for genetic manipulation by homologous recombination. All plasmids and strains used in this study are listed in Additional file 5: Table S4.

### Flow cytometry

To measure the promoter strength, the *gfp* knock-in cells were prepared in the exponential phase. Then, the cells were diluted to OD_600_ = 0.1 with 1 × phosphate-buffered saline (PBS), and the fluorescence intensity was quantitatively measured using a Guava EasyCyte flow cytometer (Millipore, Darmstadt, Germany). A total of 50,000 events were recorded to determine GFP fluorescence at a flow rate of 0.12 μL/s. The excitation was conducted by a 488-nm laser and the emission light was collected with a 525/30-nm bandpass filter.

### High-performance liquid chromatography (HPLC)

To measure cadaverine and l-lysine concentrations, 500 μL of culture supernatants was filtered through a 0.22-μm syringe filter and analysed by HPLC, as previously reported [40]. In brief, cadaverine and l-lysine concentrations were measured by precolumn *o*-phthaldialdehyde derivatization coupled with reverse-phase HPLC and UV detection. The derivatized cadaverine and l-lysine were detected by a variable wavelength detector at 230 nm.

### Statistics and reproducibility

Data obtained from at least three independent experiments were analysed using GraphPad Prism v7.0 (GraphPad Software, Inc.). The replicates were plotted using the average and standard error of the mean (SEM).

## Supplementary Information

**Additional file 1: Fig S1.** Total proteome of Methylomonas sp. DH-1 analyzed by 2D-PAGE. Five high-density protein spots are indicated by arrows and identified protein names are listed on the right.

**Additional file 2: Table S1.**Predicted promoter sequences from the genome of Methylomonas sp. DH-1.

**Additional file 3: Table S2.** Predicted promoter sequences from the genome of M. trichosporium *OB3b.*

**Additional file 4: Table S3.** Five promoter sequences identified from 2D-PAGE of *Methylomonas* sp. DH-1.

**Additional file 5: Table S4.** Bacterial strains and plasmids used in this study.

## Data Availability

Not applicable.

## Abbreviations

TCA: Tricarboxylic acid
RuMP: Ribulose monophosphate
EMP: Embden–Meyerhof–Parnas
PP: Pentose phosphate (PP)
EDD: Entener–Doudoroff
MEP: Methylerythritol 4-phosphate
2D-PAGE: 2D gel electrophoresis
MALDI-TOF: Matrix-assisted laser desorption/ionization–time of flight
PQQ: Pyrroloquinoline quinone
*mxaF*: Methanol dehydrogenase
*glnA*: Glutamine synthetase
*tuf*: Elongation factor Tu
*tal*: Transaldolase
*hps*: 3-Hexulose-6-phosphate synthase
*pmoC*: Methane monooxygenase
*gfp*: Green fluorescent protein
*NADPH*: Nicotinamide adenine dinucleotide phosphate
CadA: Lysine decarboxylase
OAA: Oxaloacetate
CadB: Lysine/cadaverine antiporter
NMS: Nitrate mineral salt
TSS: Transcription start site
PBS: Phosphate-buffered saline
