# Supplementary figures and images for: Construction of a tunable promoter library to optimize gene expression in Methylomonas sp. DH-1, a methanotroph, and its application to cadaverine production
Source: Biotechnol Biofuels. 2021 Dec 4;14:228. doi: 10.1186/s13068-021-02077-8 (PMC8645107; doi:10.1186/s13068-021-02077-8)

**Additional file 1: Figure S1.**


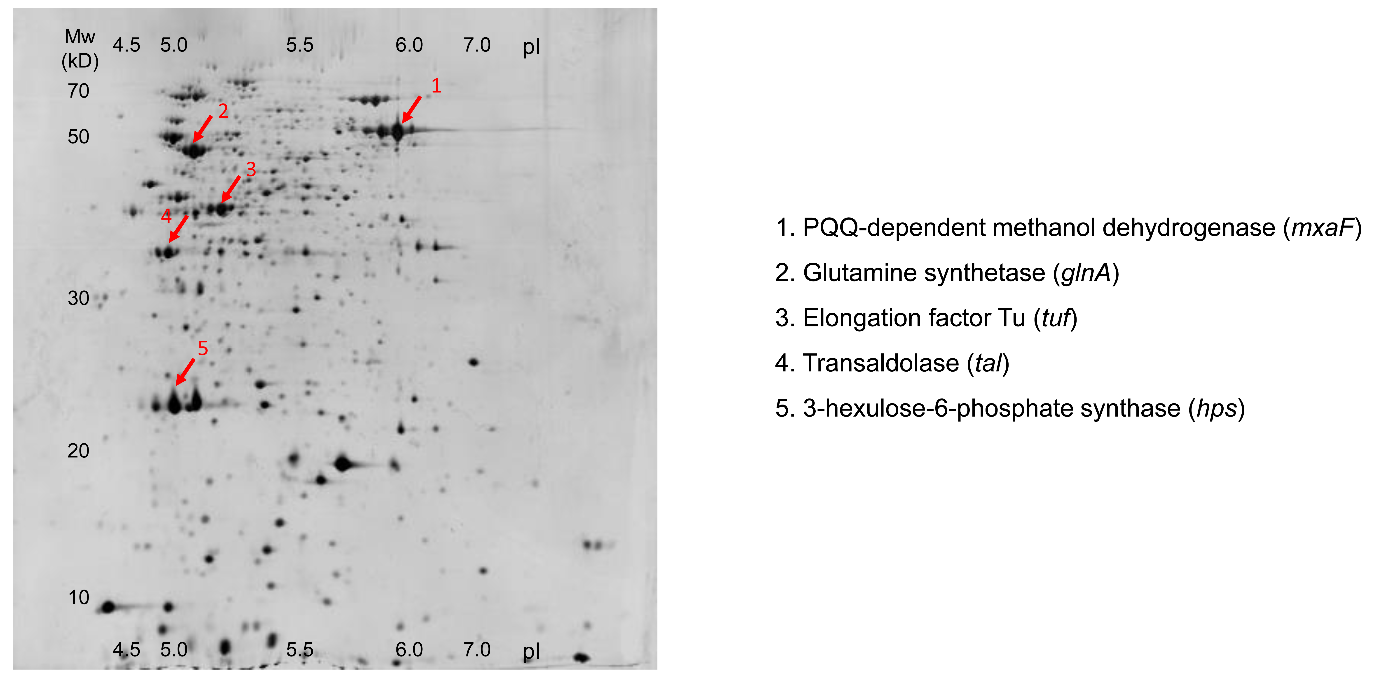

Supplement: Supplementary file 1 — Additional file 1: Fig S1. Total proteome of Methylomonas sp. DH-1 analyzed by 2D-PAGE. Five high-density protein spots are indicated by arrows and identified protein names are listed on the right. [file 13068_2021_2077_MOESM1_ESM.docx]
